# Microflow-Based Device for In Vitro and Ex Vivo Drug Permeability Studies

**DOI:** 10.1177/2472630320916190

**Published:** 2020-04-30

**Authors:** Samu Hemmilä, Marika Ruponen, Elisa Toropainen, Unni Tengvall-Unadike, Arto Urtti, Pasi Kallio

**Affiliations:** 1 Faculty of Medicine and Health Technology, Tampere University, Tampere, Finland; 2 School of Pharmacy, Faculty of Health Sciences, University of Eastern Finland, Kuopio, Finland; 3 Division of Pharmaceutical Biosciences, Faculty of Pharmacy, University of Helsinki, Helsinki, Finland; 4 Laboratory of Biohybrid Technologies, Institute of Chemistry, St. Petersburg State University, St. Petersburg, Russian Federation

**Keywords:** permeability, in vitro, ex vivo, microfluidics

## Abstract

This paper presents a novel microflow-based concept for studying the permeability of in vitro cell models or ex vivo tissues. Using the proposed concept, we demonstrate how to maintain physiologically relevant test conditions and produce highly reproducible permeability values for a range (31) of drug compounds. The apparent permeability coefficients (*P*_app_) showed excellent correlation (0.89) with the values from experiments performed with a conventional Ussing chamber. Additionally, the microflow-based concept produces notably more concentrated samples than the conventional Ussing chamber-based approach, despite the fact that more than 10 times smaller quantities of test compounds and biological membranes are needed in the microflow-based concept.

## Introduction

Absorption, distribution, metabolism, and excretion (ADME) studies are commonly used for screening the most suitable molecules in drug development.^1^ Besides intravascular (IV) administration, all other routes of administration require adsorption of the drug molecules from the site of administration to the bloodstream to have any therapeutic effect.^2^ This includes, for example, orally or topically administered medicines, which are more convenient routes of administration than IV delivery.^2,3^

Permeability across a biological membrane is one of the factors affecting the absorption and distribution of drugs. It can be studied using animals (in vivo), excised tissues (ex vivo), and cell monolayers or artificial membranes (in vitro), and with computational methods (in silico).^1–4^ In general, the research method is selected based on the number of molecules being studied:^5^ in silico approaches can analyze thousands of compounds relatively fast, and thereafter, in vitro and ex vivo experiments can be used to test a smaller number of selected compounds. Before entering clinical trials, many compounds are tested using in vivo animal experiments. However, there is an increasing ethical^6^ and legislative (e.g., the REACH regulation in Europe) demand to replace, reduce, and refine animal experiments using in vitro or in silico methods.

The conventional devices for in vitro or ex vivo permeability studies include well plates with hanging cell inserts (Transwell Assay),^7–9^ and different Ussing chamber devices.^10,11^ However, the use of the Transwell Assay is limited to cells, which can be cultured in the Transwell inserts possessing a fixed base membrane, most commonly a polycarbonate (PC) or a polyethylene terephthalate (PET) membrane. It is also known that the Transwell Assay, as well as other static devices, suffers from poorly controlled hydrodynamic diffusion layers at the membrane surface.^12,13^ The Ussing chamber supports the use of different base membranes including ex vivo tissues, but it shares the potential problems related to the hydrodynamic diffusion layers, whose intertest differences in the thickness may affect the measured permeability values. Additionally, the use of an Ussing chamber may lead to cross-contaminations caused by its reusable acrylic components.^10^

Many of the conventional devices handle static fluid volumes, which may be rather large. The use of large and static fluid volumes results in a need for high amounts of test molecules in the donor side, to achieve measurable concentrations in the receiver side, within a reasonable timescale. This may exclude the use of physiologically relevant conditions, which may decrease the relevance of the results. In addition to molecule concentrations, in vivo*-*like test conditions include other parameters, such as the temperature, pH, and membrane surface area.^8,10,14^ Temperature influences the permeability of both in vitro^14^ and ex vivo^15^ membranes, and also pH plays a role in the permeability of molecules.^16^ In addition, the integrity of the membrane may be affected by the pH of the liquids being in contact. Despite their importance, the temperature and pH are often inadequately controlled in devices that are used outside an incubator.

To avoid the limitations of large and static volumes, different microfluidic chips for permeability studies have been developed.^17–20^ Typically, such chips do not support the use of ex vivo tissues but are tailored for a certain cell type. The cells must be cultured within the chips, most commonly made of polydimethylsiloxane (PDMS). However, PDMS has limited reusability possibilities, and it is known for its tendency for unspecific molecular adsorption, making the chips unusable at low molecule concentrations.^21–24^ Furthermore, the correct phenotype of the cultured cells in the PDMS chip is uncertain, and should always be verified. Thus, the use of microfluidic chips has not yet gained wide acceptance in membrane permeability studies.

This article presents a novel concept for in vitro and ex vivo permeability studies. The proposed concept was developed to overcome the shortcomings in both conventional devices and microfluidic chips, and therefore (1) it can be used to study the permeability of any in vitro epithelial cell model or ex vivo tissue sample; (2) it provides low molecule and cell/tissue consumption; (3) it allows the study of low drug quantities and concentrations, due to its microfluidic volumes and material selection; (4) consumable components of the concept can be cost-effectively injection molded, which enables their disposable use and thus eliminates the risk of cross-contamination; (5) it includes a tabletop control unit, which has been demonstrated to accurately mimic in vivo experiment conditions; and (6) this study also demonstrates that the concept produces repeatable permeability values, which correlate well to those obtained with an Ussing chamber.

## Materials and Methods

### Working Principle

**Figure 1.**
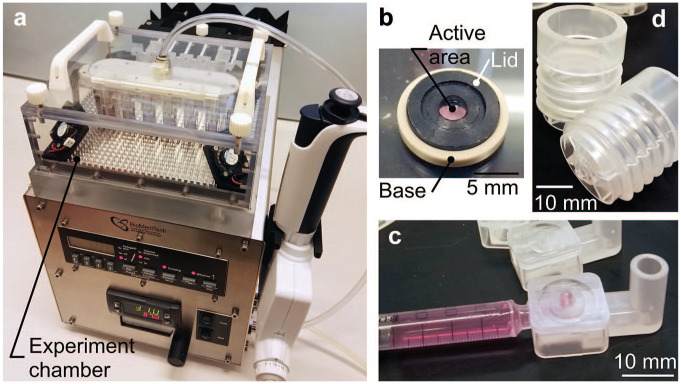
The device developed for in vitro and ex vivo permeability studies includes (**a**) a control unit, and disposable plastic components called (**b**) a membrane holder, (**c**) a flow component, and (**d**) a donor component. The specific environment used in an experiment is generated inside an experiment chamber of the control unit, where the disposables are in contact with the studied molecules and membranes.

The device used in the proposed concept is presented in **Figure 1**. It consists of a reusable control unit (**Fig. 1a**) and disposable plastic components. The disposables include a membrane holder (**Fig. 1b**), a flow component (**Fig. 1c**), and a donor component (**Fig. 1d**), and only these components are in direct contact with the fluids and samples used in the study. This eliminates the risk of cross-contamination. The control unit is responsible for generating proper and stable test conditions inside an experiment chamber, into which the disposables are placed. One environment chamber supports up to six parallel permeability experiments at a time.

**Figure 2.**
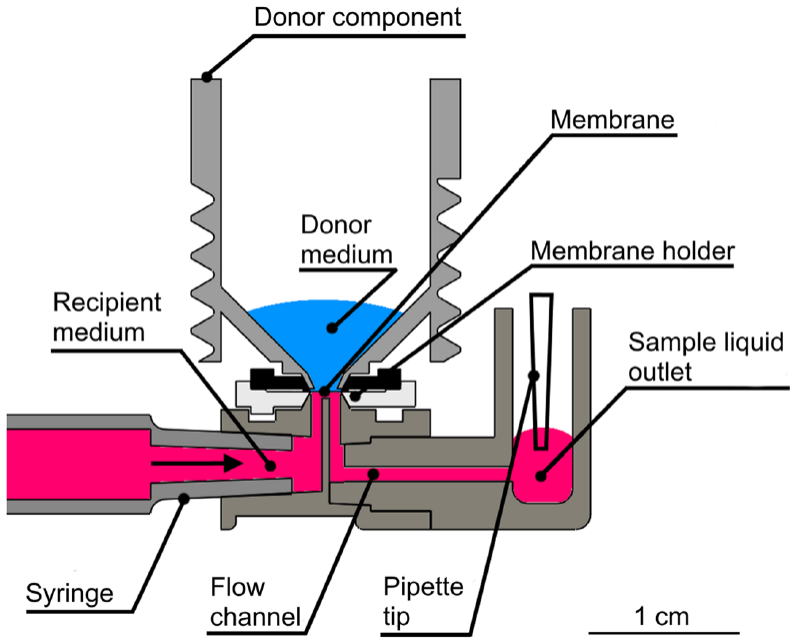
Schematic presentation of one of the six parallel permeability experiments inside the experiment chamber, in a controlled and stable environment. The membrane being studied is placed into the membrane holder, on top of which is the donor component containing a donor medium. The donor medium includes the drug molecules under study. The molecules penetrate the membrane and enter a recipient medium, after which the flowing recipient medium transports the molecules through the flow channel to the sample liquid outlet, from where the sample is collected and analyzed.

A schematic presentation of a permeability experiment taking place inside the environment chamber is shown in **Figure 2**. As the figure illustrates, the membrane being studied is placed in the membrane holder, which enables the study of any in vitro cell model cultured on a semipermeable membrane, or virtually any type of artificial or ex vivo tissue membrane. The drug or other molecules being studied are applied to the donor component, which is placed onto the membrane holder. The flow component with the flowing recipient medium is located below the membrane holder. During the permeability study, the molecules from the donor component penetrate through the membrane and enter the recipient medium. The flow of the recipient medium carries the molecules through a flow channel to the liquid outlet, which simultaneously promotes the permeation of new molecules by lowering the sample concentration beneath the membrane, and thus maintaining a desired concentration gradient. The low recipient medium volume beneath the membrane keeps dilution of the sample liquid minimal and also enables the study of low drug concentrations, widening the application potential of the device. The samples are collected from the sample liquid outlet, after which the molecule concentrations of the collected samples can be analyzed using, for example, mass spectrometry (MS). One permeability experiment consumes a single item of each disposable component.

### Components of the Device

The control unit, shown in **Figure 1a**, is used to generate and maintain the desired temperature, pH, and flowrate of the recipient medium in a permeability study. These three parameters are typically set to mimic the in vivo conditions. A more detailed description of the control unit and its functions is included in the supporting information.

The most important design targets for the disposables were (1) the enablement of study of different in vitro, ex vivo, or artificial membranes and (2) the use of low-molecule concentrations in the donor side. In addition, the final geometry of the components was influenced by the requirement of disposability, which demanded injection molding as a fabrication method to keep manufacturing costs low. To produce both functional and disposable components cost-effectively, a large set of raw materials were also studied as described in the supporting information.

The membrane holder (**Fig. 1b**) was developed to answer the need for a versatile membrane selection. It consists of two components: a lid and a base. The membrane being studied is placed on the base component, after which the holder is closed by fixing the lid in the base and onto the membrane using a closing tool. The membrane holder has four designs for different membrane thicknesses. Each holder design covers a membrane thickness range of approximately 0.5 mm, depending also on the compression of the membrane. Thus, artificial or biological membranes having thicknesses ranging from micrometers to approximately 2 mm can be studied using the holder. To keep the sample consumption low, the holder has an active area of only 3.1 mm^2^, meaning the area of the membrane surface in contact with the donor and recipient mediums.

The holder can be used for culturing of in vitro membranes, or with any in vitro membranes cultured elsewhere (e.g., in Transwell inserts). In the latter case, the membranes, such as cell monolayers, cultured on a semipermeable supporting material are inserted into the holders before a permeability experiment, and the holders with the membranes are then preserved in a commercial 12-well plate in a conventional incubator. Both culturing and the use of transplanted membranes allow preparation of a large number of membranes at once. Before starting a permeability experiment, the integrity of the membrane (both in vitro and ex vivo) is ensured by performing transepithelial electrical resistance (TEER) measurements as described in the supporting information. The geometrical design of the holder also allows for visual inspection of the membrane under a microscope.

The flow component presented in **Figure 1c** facilitates the use of low-recipient-medium volumes. The low volumes, combined with the use of a flowing recipient medium, enables the use of low concentrations in the donor medium. The flow component includes (1) a connector for a disposable syringe, (2) a connector pad for the sample holder, (3) a liquid sample outlet that stores the collected sample liquid, and (4) a mini-channel connecting these four features, as illustrated in **Figure 2**. The mini-channel was designed to have approximately 1 µL of recipient medium volume, which makes the dilution of the drug molecules penetrating through the membrane minimal.

The donor component is shown in **Figure 1d**, and it is capable of reserving 2 mL of donor medium. Due to the design of the donor component, low donor medium volumes down to approximately 10 µL can be studied. This helps if the availability of the molecules is limited, such as in the case of biological drugs.

### Validation of the Control Unit

The performance of the control unit in maintaining the desired temperature, pH, and flowrate was studied to validate its ability to mimic physiological conditions. A Sensirion (Staefa ZH, Switzerland) SLI liquid flow meter was used for monitoring the flowrate of the recipient medium. The flowrate was set to 1.0 µL/min, and it was studied for approximately 2 h.

To quantify the temperature in the relevant area of the control unit, that is, in the liquids and close to the membrane, an additional thermal sensor was placed inside a disposable syringe attached to the flow component. The uniformity of the temperature distribution was studied using a Fluke (Everett, WA) Ti400 thermal camera. The temperature measurement using the sensor was started after a 25 min stabilization period, and the temperature data were collected for 10 h. The temperature was set to 37.0 °C.

Dulbecco’s Modified Eagle Medium/Nutrient Mixture F-12 (DMEM/F-12; Thermo Fisher, Waltham, MA) with 1% penicillin-streptomycin was used in the pH study. The medium was stored in a conventional incubator prior to the experiment. The temperature was set to 37.0 °C, to mimic the average normal body temperature. The flowrate of the gas (5% CO_2_, 19% O_2_, 76% N_2_) was set to 100 mL/min, after which 2 mL of the medium was pipetted to each donor component. The pH values of all six donor components were studied for 6 h, and the measurements were taken at 1 h intervals using a Sentron (VD Leek, The Netherlands) SI pH meter.

### Permeability Study

Permeability studies were performed with isolated porcine cornea and a cassette mix containing 31 clinically used drug molecules (**Table 1**) covering a large chemical space.^25,26^ The preparation of the cassette mix and the isolation of the porcine cornea were performed as described by Ramsay et al.^25,26^

**Table 1.** Drug Molecules in the Cassette Mix.^25^

Acetazolamide	Betaxol	Diclofenac	Levocobastine	Pilocarpine
Acyclovir	Brinzolamide	Dorzolamide	Lincomycin	Pindolol
Ampicillin	Bromfenac	Fluconazole	Lornoxicam	Prednisolone
Atropine	Carteolol	Ganciclovir	Methazolamide	Propranolol
Atenolol	Ciprofloxacin	Indomethacin	Methotrexate	Quinidine
Aztreonam	Dexamethasone	Ketorolac	Nadolol	Tizanidine
				Voriconazole

In the permeability experiments, the temperature was set to 35.0 °C, to mimic the physiological temperature of cornea. Porcine cornea tissues were placed in the membrane holders, and the TEER values of the membranes were measured. The temperature in the chamber was left to stabilize for approximately 1 h. The membrane holders containing the cornea and the other disposable components were fastened to the environment chamber. The cassette mix (500 µL) was pipetted into the six donor components, and a CO_2_ gas flow of 100 mL/min was supplied to the donor components. A flow of the recipient medium at a flowrate of 1.0 µL/min was set for the syringe pump. A balanced salt solution (BSS Plus; Alcon Laboratories, Fort Worth, TX) was used as the recipient medium. An initialization period of 30 min was used to guarantee that each mini-channel was completely filled with the recipient medium. After that, all the sample liquid outlets were emptied using a six-channel pipette in order to ensure identical starting points for each parallel permeability experiment. Then, a 6 h permeability study was started. During the study, 30 µL samples were collected from each sample outlet at 30 min intervals, yielding a total of 12 collected samples for each membrane. The samples were stored at −20 °C prior to analysis. The permeability study was replicated to check the repeatability of the results, and three vertical Ussing chambers connected to a voltage–current clamp, as described by Ramsay et al.,^26^ were used in parallel with the microflow-based device, to produce comparative permeability data.

The drug concentrations of the collected samples were analyzed using a liquid chromatography–tandem mass spectrometry (LC-MS/MS) system as described by Ramsay et al.^25^ The determined concentrations were used to calculate the cumulative masses of the cornea-permeated drug molecules, which were further utilized for calculation of the apparent permeability coefficient (*P*_app_) values, using eq 1:

(1) $$P_{\mathrm{app}}=J/\left(C_{0}A\right)$$

In the equation, *J* (ng/s) is the drug flux (linear range) across the tissue, *C*_0_ is the initial donor concentration (ng/cm^3^), and *A* is the area of the exposed tissue (cm^2^).

## Results and Discussion

### Validation of the Control Unit

The validation experiment demonstrated that the control unit enables permeability experiments that mimic a physiological environment accurately. The average temperature in the most relevant area of the environment chamber was measured to be 36.6 °C, which was only 0.4 °C off the target value. The standard deviation of the temperature value was 0.8 °C, and the temperature also had excellent uniformity (± 0.4 °C) in the chamber as shown by the thermal image map presented in the supporting information. The flowrate measurement showed an average flowrate of 1.1 µL/min (± 0.1 µL/min) for the recipient medium. The pH was kept precisely at the target value during the 6 h experiment, as the average of the first measurement was 7.42, after which averages of between 7.42 and 7.47 were measured. The validation results are combined in **Table 2**, and a diagram illustrating the measured pH values is included in the supporting information.

**Table 2.** Summary of the Control Unit Validation.

	Temperature (°C)	Flowrate (µL/min)	pH
Target value	37.0	1.0	7.46
Measured (average ± SD)	36.6 ± 0.4	1.1 ± 0.1	7.45 ± 0.02

### Permeability Study

The porcine cornea tissues used in the permeability study demonstrated good integrity, as the measured TEER values were 227 ± 64 Ω × cm^2^. Comparison values for the tissues used in the Ussing chamber were 372 ± 54 Ω × cm^2^.

**Figure 3.**
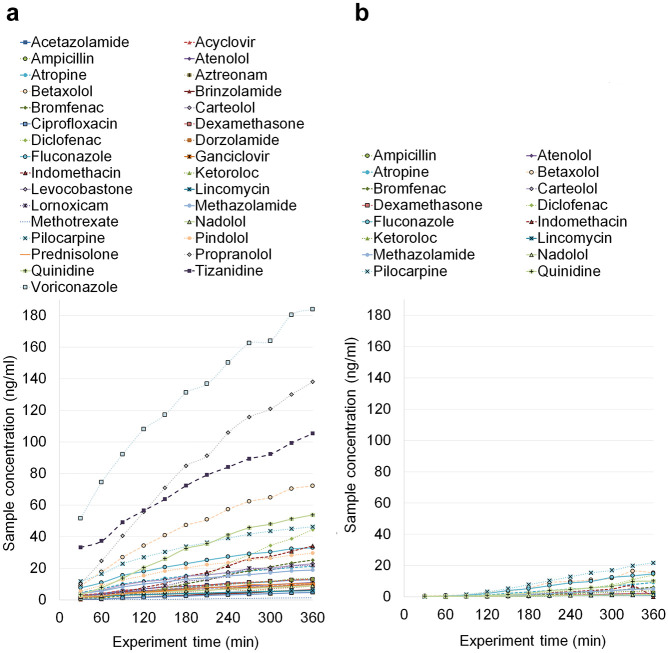
The mean drug concentration in the receiver side as a function of time (**a**) in the microflow-based device and (**b**) in the Ussing chamber. The number of parallel tissues was 5–11 and 3 in the studies with the microflow device and the Ussing chamber, respectively.

The drug concentrations for the drug compounds in the receiver side as a function of time are presented in **Figure 3a** for the microflow-based device, and comparison data for the Ussing chamber in **Figure 3b**. It is clearly visible that the microflow-based device produced notably more concentrated samples than the Ussing chamber, which only produced analyzable (concentration above the limit of quantification) samples for 16 molecules (**Fig. 3b**). This is despite the fact that one Ussing chamber unit consumed 13 times more (6.5 vs 0.5 mL) donor solution and had almost 21 times higher membrane surface area (3.1 and 63.6 mm^2^). This indicates the high potential for the microflow-based system to study low drug concentrations and to save possibly limited or expensive tissue membranes.

**Figure 4.**
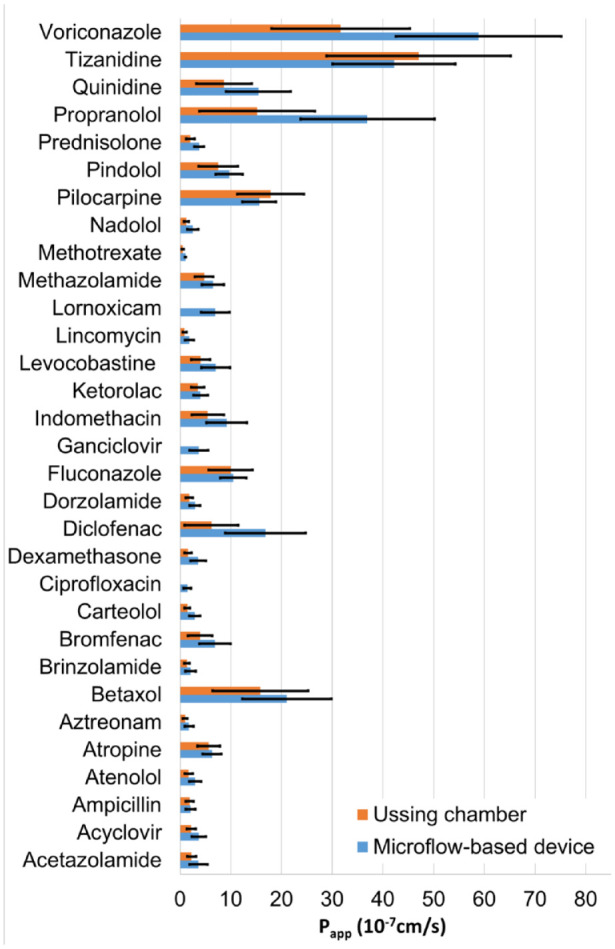
Apparent permeability coefficient (*P*_app_) values for porcine cornea obtained with the microflow-based device (*n* = 5–11 ± SD) and the Ussing chamber (*n* = 2–7 ± SD; Ramsay et al.^26^).

**Figure 5.**
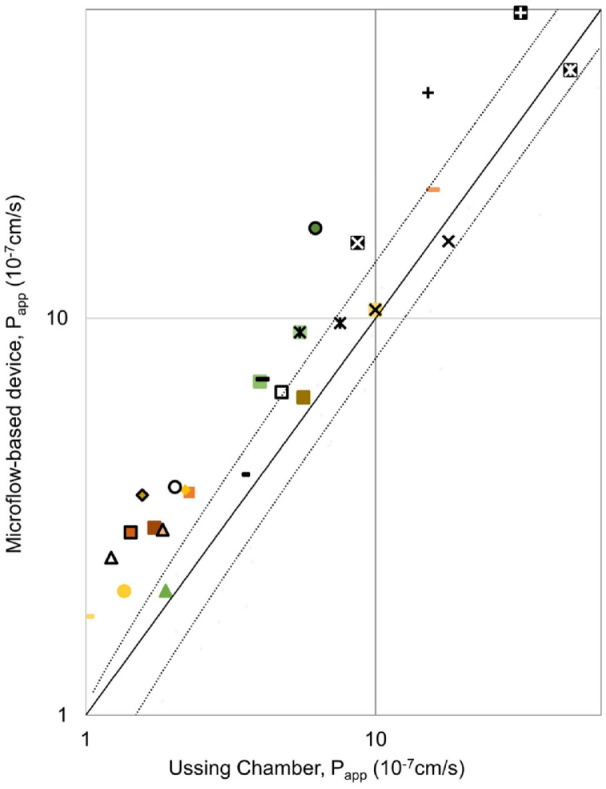
Correlation between the *P*_app_ values obtained by the microflow-based device and the Ussing chamber. The values with the microflow-based device are on average 1.65 ± 0.45 times higher than the ones obtained with the Ussing chamber. This is illustrated in the figure by the black trend lines.

The calculated *P*_app_ values are presented for both methods in **Figure 4**. In **Figure 4**, Ussing chamber data by Ramsay et al.^26^ were combined with the results obtained in this study, to increase the number of comparable drug molecules. It is notable that Ramsay et al.^26^ used a two times higher exposure concentration in order to obtain detectable drug concentrations in the receiver side. The drug permeability values obtained by both methods showed over 50-fold variation (**Fig. 4**). This variation is explained by the tight barrier properties of the cornea and the different physicochemical properties of the drug molecules (Ramsay et al.^25^). Despite the notable differences in the collected drug concentrations in the receiver side (**Fig. 4**), the *P*_app_ values from the microflow-based device and the Ussing chamber show excellent correlation (**Fig. 5**). This is understandable since the permeability coefficient, as opposed to drug flux across the membrane, is not dependent on the drug concentration in the donor side. This correlation was further confirmed by calculating Pearson’s correlation coefficient, 0.89.

Interestingly, the *P*_app_ values from the microflow-based device were shown to be consistently higher than the values obtained with the Ussing chamber (**Fig. 5**). The difference is 1.65 times on average, with a standard deviation of 0.45.

The main reason for the higher concentrations, and thus consistently higher *P*_app_ values of the microflow-based device may be the thicker static diffusion layers in the Ussing chamber. Other possible factors include the raw material of the Ussing chamber (polymethyl methacrylate [PMMA]), which was shown to adsorb molecules, and the uncontrolled molecule gradient of the device. The differences in the measured TEER values of the tissues may also affect the results.

## Conclusions

This paper proposes a microflow-based approach for studying the molecular permeability of cell monolayers, artificial membranes, and excised tissues. The paper demonstrates that the proposed concept maintains physiologically relevant test conditions and produces highly reproducible permeability values for a range (31) of drug compounds. Moreover, it produces notably more concentrated samples than the conventional reference method with a 13 times lower volume of test compounds and a 21 times smaller surface area of the biological membranes.

In addition to reliable permeability testing, this microflow-based device may also be used in other applications. The membrane holder allows the study of virtually any kind of tissue or artificial sample, and the structure of the environment chamber enables the studied membranes to be exposed to different liquids or gases, whose flowrate can be accurately controlled. Example applications include toxicology and safety studies, skin exposure experiments, perfusion-based cell culturing, and inhalation toxicology studies.

## Supplemental Material

Supplemental_Material_for_Microflow-Based_Device_for_in_Vitro_and_ex_Vivo_drug_Permeability_Studies_by_Hemmila,_et_al – Supplemental material for Microflow-Based Device for In Vitro and Ex Vivo Drug Permeability Studies Click here for additional data file. Supplemental material, Supplemental_Material_for_Microflow-Based_Device_for_in_Vitro_and_ex_Vivo_drug_Permeability_Studies_by_Hemmila,_et_al for Microflow-Based Device for In Vitro and Ex Vivo Drug Permeability Studies by Samu Hemmilä, Marika Ruponen, Elisa Toropainen, Unni Tengvall-Unadike, Arto Urtti and Pasi Kallio in SLAS Technology
