# Supplementary material for: Microflow-Based Device for In Vitro and Ex Vivo Drug Permeability Studies
Source: SLAS Technol. 2020 Apr 30;25(5):455–62. doi: 10.1177/2472630320916190 (PMC7509603; doi:10.1177/2472630320916190)
Supplement: Supplemental_Material_for_Microflow-Based_Device_for_in_Vitro_and_ex_Vivo_drug_Permeability_Studies_by_Hemmila,_et_al – Supplemental material for Microflow-Based Device for In Vitro and Ex Vivo Drug Permeability Studies [file Supplemental_Material_for_Microflow-Based_Device_for_in_Vitro_and_ex_Vivo_drug_Permeability_Studies_by_Hemmila,_et_al.pdf]

Supplemental Material

Microflow-based Device for *in Vitro* and *ex Vivo* Drug Permeability Studies

Samu Hemmilä[*,1], Marika Ruponen[2], Elisa Toropainen[2], Unni Tengvall-Unadike[2], Arto Urtti[2,3,4], Pasi Kallio[1]

[1]Faculty of Medicine and Health Technology, Tampere University, 33720 Tampere, Finland

[2]School of Pharmacy, Faculty of Health Sciences, University of Eastern Finland, 70211 Kuopio, Finland

[3]Division of Pharmaceutical Biosciences, Faculty of Pharmacy, University of Helsinki, 00014 Helsinki, Finland

[4]Laboratory of Biohybrid Technologies, Institute of Chemistry, St. Petersburg State University, 198540 St Petersburg, Russian Federation

Keywords: Permeability, *in vitro*, *ex vivo*, microfluidics

*corresponding author: Samu Hemmilä, Faculty of Medicine and Health Technology, Tampere University, Korkeakoulunkatu 7, 33720 Tampere, Finland. Email: samu.hemmila@tuni.fi

Control Unit

The temperature inside the experiment chamber is adjusted by using a proportional-integral-derivative (PID) controlled heating element, which is located in the bottom of the chamber. An aluminum heat sink and a fastening slider for the disposables are placed on top of the heating element. Four fans in the chamber promote a uniform temperature distribution within the chamber and over the disposable components. The sink, the slider and the fans in the experiment chamber are illustrated in Figure S1. In addition, the experiment chamber contains a temperature sensor, which provides temperature information for the PID controller and the user.

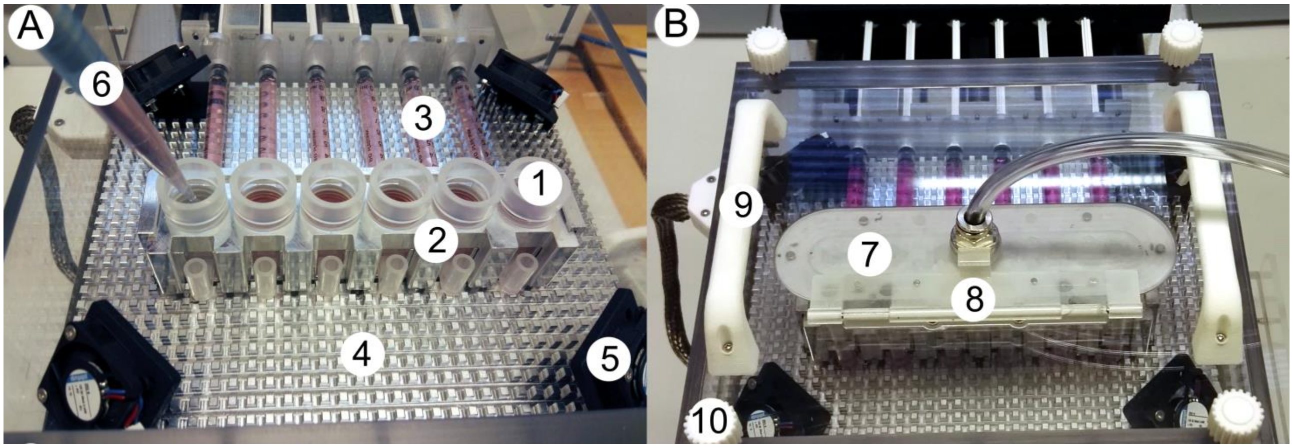

Figure S1. a) The environment chamber without a lid: The disposables (1) are fastened to a slider component (2) and connected to the syringes (3) containing the recipient medium. A heating element combined with a heat sink (4) and fans (5) in each corner of the chamber stabilize the temperature inside the chamber. The donor medium (6) is pipetted into the donor components. b) The lid of the experiment chamber includes a CO_2 feeder (7), which distributes the gas onto each donor component and stabilizes the pH of the donor medium. A sample hatch (8) is used to collect the sample liquid, and handles (9) as well as thumb screws (10) are used to close the chamber tightly.

The pH is controlled by supplying CO_2 gas into the donor medium. The CO_2 feeder, visible in Figure S2b, distributes the gas into each of the six donor components through a gas-permeable membrane. The membrane reduces the evaporation of the donor medium. The CO_2 gas is supplied from an external cylinder, which is connected to the side of the casing using a tube, as seen in Figure S2a.

The flow of the recipient medium is generated by using a syringe pump mechanism, depicted in Figure S1a, and disposable syringes connected to the flow component, also visible in Figure S1a. The set-points for the flowrate of the recipient medium, the temperature and the CO_2 flowrate are controlled through the front panel of the control unit, which is presented in Figure S2b. The panel also displays the current and set values for the temperature and the gas flowrate, and the set value for the liquid flowrate.

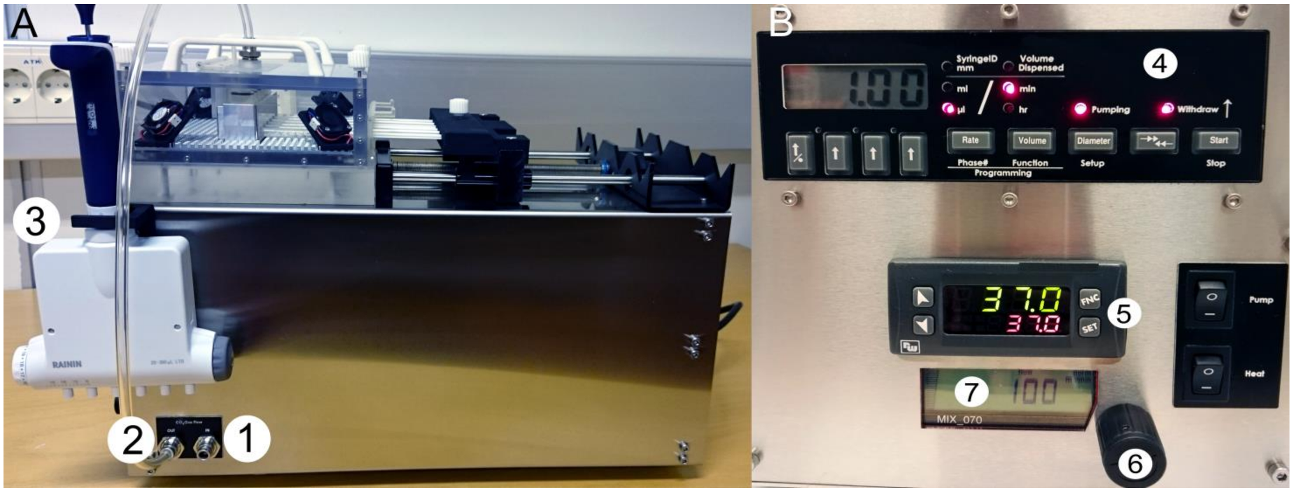

Figure S2. a) The side of the control unit includes inlet (1) and outlet (2) connectors for CO_2 gas, and a holder (3) for the six-channel pipette for the liquid sample collection. b) The top panel (4) on the front of the unit is used for controlling the flowrate of the recipient medium. The middle panel (5) is a PID controller for the temperature that also displays the current measured temperature. A control knob (6) and a display (7) for the flowrate are located at the bottom.

The liquid samples for analyzing the amount of permeated drug are collected via a sample hatch, using a commercial 6-channel pipette (Mettler Toledo, Columbus, Ohio). The hatch is visible in Figure S1b, and it allows sample collection without disturbing the experiment conditions. Besides the hatch and the CO_2 feeder, the lid of the experiment chamber includes handles and thumb screws for sealing the chamber tightly. The material of the experiment chamber is polycarbonate (PC), and the casing of the unit was made of stainless steel. The size of the unit is 25 x 33 x 43 cm, and thus it only takes up a little space on a laboratory table.

TEER Measurements

A 12-well adapter, presented in Figure S3a, was developed for the TEER measurements. When an adapter containing the holder is placed on a 12 well plate, the adapter divides separate liquid volumes above and below the membrane, as illustrated in Figure S3b. Then, a TEER measurement device equipped with a commercial chopstick electrode can be used for the measurements. After the measurement, a removing tool (Figure S3) was developed to remove the holder from the adapter.

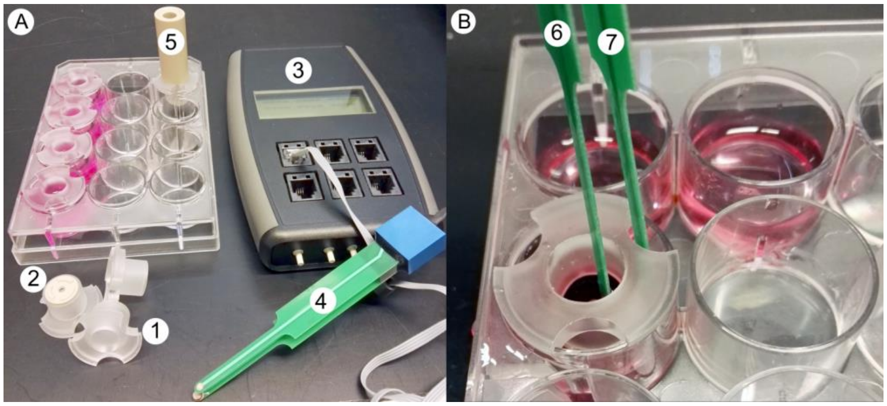

Figure S3. a) The 12-well plate adapter (1) enables TEER measurements directly from the membrane holder (2) using conventional TEER measurement devices (3) equipped with, for example, a commercial chopstick electrode (4). A tool for removing the holder from the adapter (5) was also developed. b) TEER values are measured by placing the electrodes into the adapter, and then onto the holder (6) and into the well (7).

Raw material studies

To eliminate the risk of reuse, and thus cross-contamination, the disposable components had to be made of a material that was both cheap to buy, and easy to fabricate. Additionally, the material could not excessively adsorb the molecules being studied, which would have also influenced the results of the permeability experiments. Therefore, the adsorption properties of thermoplastic polymers which are cost-effective to mass manufacture by injection molding were studied in order to find suitable materials for the disposables.

These experiments included seven polymers, namely cyclo-olefin polymer (COP), glycol-modified polycyclo-hexylenedimethylene terephthalate (PCTG), high density polyethylene (PE-HD), polyacetal

(POM), polymethyl meth-acrylate (PMMA), polystyrene (PS) and PP. The adsorption of these materials was studied using highly lipophilic Nile Red (logP = 5.0)[22], which has earlier been shown to be adsorbed onto PDMS[23]. PDMS was thus selected as the negative reference in this study, and glass as a positive reference[24]. In the staining protocol, the material samples were immersed in Nile Red (Sigma Aldrich, Saint Louis, Missouri, 0.03 mg/ml in 20 % EtOH) for 30 minutes, after which they were rinsed using deionized (DI) water, and imaged using a Zeiss (Oberkochen, Germany) fluorescence microscope. The images were recorded using an Alexa 546 filter, four-times magnification and an exposure time of 430 ms.

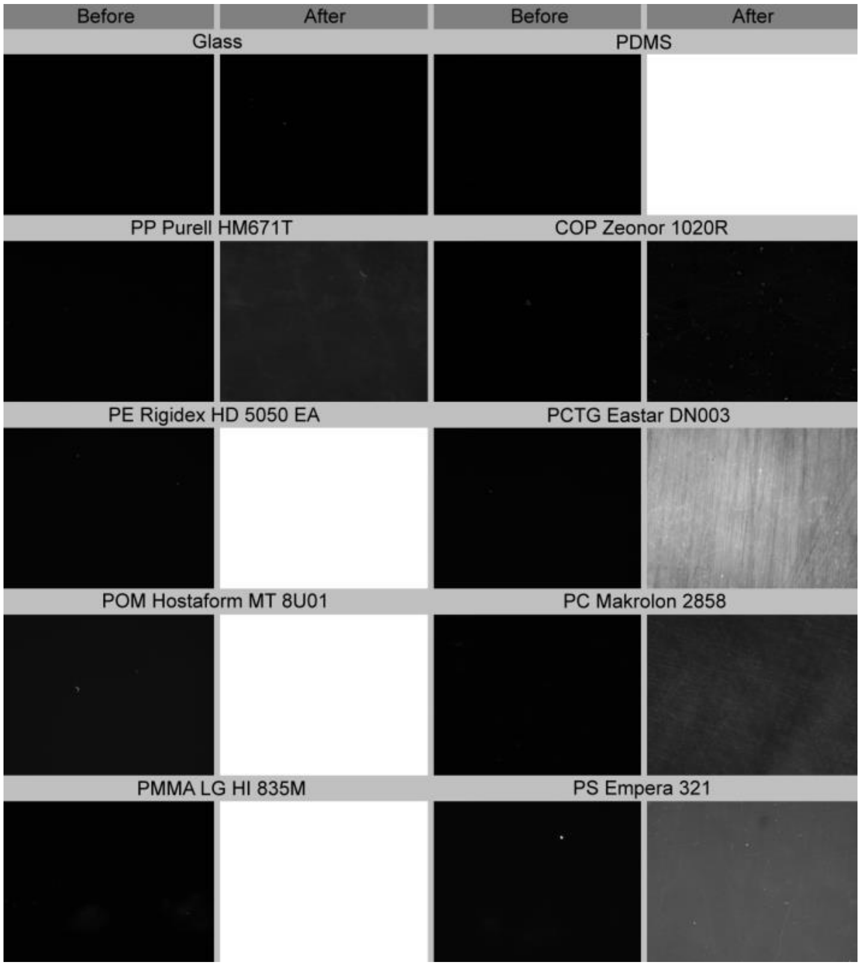

Figure S4. The adsorption of Nile Red into different materials imaged by a fluorescence microscope. The darker colors revealed that PP, COP and PC did not notably adsorb Nile Red molecules, similarly to glass. In addition, PDMS, PMMA, PE and POM adsorbed excessive amount of molecules. PCTG and PS only experienced minimal molecule adsorption.

The results of the material validation experiment are illustrated in Figure S4, where a lighter color represents the unwanted adsorption of Nile Red molecules. The results showed that PP, COP and PC exhibited desirable adsorption properties similar to glass i.e. their adsorption of Nile red was negligible. PE, POM and PMMA exhibited weaker properties, similar to PDMS. PS and PCTG were also shown to adsorb Nile red, but not as much as PDMS.

Based on the adsorption experiment, PC and PP were selected as the materials for the disposables, since they are more easily available than COC. Two materials having different hardness values were selected to promote leak-free sealing between the disposables. Therefore, the donor components and the flow components were made from the softer PP, while the PC was used for the membrane holder. However, this study used a prototype series of holders, which were manufactured from polyetheretherketone (PEEK). PEEK was selected as the prototype material due to its suitability for precise machining and its chemical inertness.

Validation of the Control Unit

The temperature distribution inside the control unit is shown in Figure S5, and Figure S6 presents the results of the pH study included in the validation experiment of the control unit.

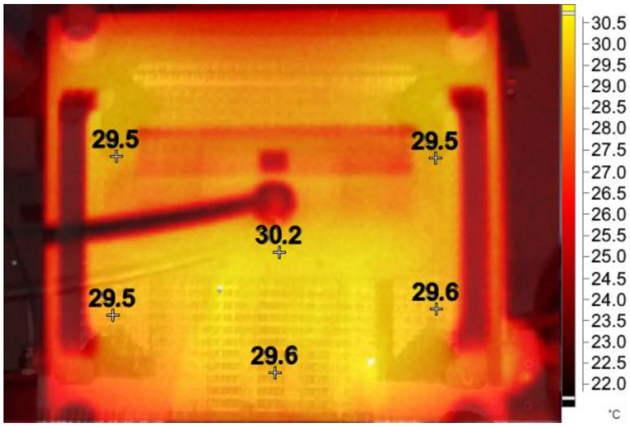

Figure S5. A thermal camera was used to study the temperature distribution in the environment chamber. It should be noted that the image does not reveal the absolute temperature value inside the chamber (36.6 °C on average).

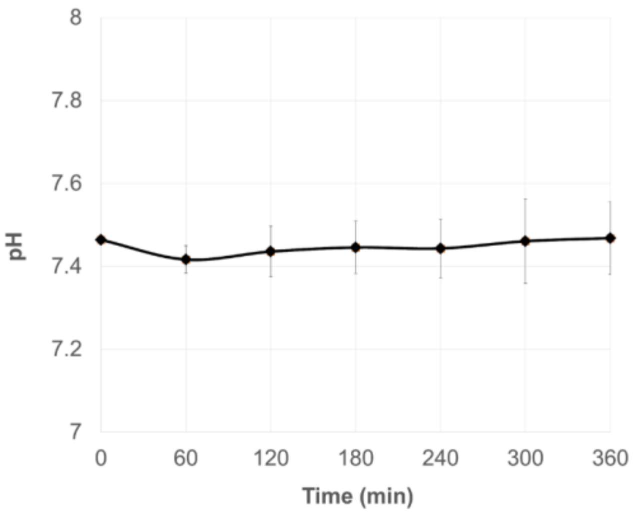

Figure S6. Average pH value of the six donor components as a function of time. Standard deviation bars are also included.
